# AFM-compatible microfluidic platform for affinity-based capture and nanomechanical characterization of circulating tumor cells

**DOI:** 10.1038/s41378-020-0131-9

**Published:** 2020-03-23

**Authors:** Muhammedin Deliorman, Farhad K. Janahi, Pavithra Sukumar, Ayoub Glia, Roaa Alnemari, Samar Fadl, Weiqiang Chen, Mohammad A. Qasaimeh

**Affiliations:** 1 Division of Engineering, New York University Abu Dhabi, P.O. Box 129188, Abu Dhabi, UAE; 2 Mohammed Bin Rashid University of Medicine and Health Sciences, P.O. Box 505055, Dubai, UAE; 3 Department of Mechanical and Aerospace Engineering, New York University, New York, NY 10003 USA; 4 Division of Science, New York University Abu Dhabi, P.O. Box 129188, Abu Dhabi, UAE; 5 Department of Biomedical Engineering, New York University, New York, NY 10003 USA

**Keywords:** Biosensors, Chemistry

## Abstract

Circulating tumor cells (CTCs) carried by the patient’s bloodstream are known to lead to the metastatic spread of cancer. It is becoming increasingly clear that an understanding of the nanomechanical characteristics of CTCs, such as elasticity and adhesiveness, represents advancements in tracking and monitoring cancer progression and metastasis. In the present work, we describe a combined microfluidic–atomic force microscopy (AFM) platform that uses antibody–antigen capture to routinely isolate and nanomechanically characterize CTCs present in blood samples from prostate cancer patients. We introduce the reversible assembly of a microfluidic device and apply refined and robust chemistry to covalently bond antibodies onto its glass substrate with high density and the desired orientation. As a result, we show that the device can efficiently capture CTCs from patients with localized and metastatic prostate cancer through anti-EpCAM, anti-PSA, and anti-PSMA antibodies, and it is suitable for AFM measurements of captured intact CTCs. When nanomechanically characterized, CTCs originating from metastatic cancer demonstrate decreased elasticity and increased deformability compared to those originating from localized cancer. While the average adhesion of CTCs to the AFM tip surface remained the same in both the groups, there were fewer multiple adhesion events in metastatic CTCs than there were in their counterparts. The developed platform is simple, robust, and reliable and can be useful in the diagnosis and prognosis of prostate cancer as well as other forms of cancer.

## Cancer research: Investigating the drivers of metastasis

A microfluidic platform allows for the capture and characterization of circulating tumor cells, a major driver of invasive cancer. Circulating tumor cells (CTCs) break off from the original tumor and invade distant sites; however, they’re hard to isolate and analyze because their concentration in blood is so low. Mohammad Qasaimeh, of New York University Abu Dhabi, and his team of UAE and US scientists created a device with microfluidic channels, that traps cancer cells using targeted antibodies that are bound to the device’s glass surface. Once captured, a high-resolution microscopy technique can measure the cells’ mechanical attributes. The team tested the device on prostate cancer cells and describe that CTCs from metastatic cancers show differences in elasticity, deformability, and adhesion when compared to localized cancers. This technique offers a promising tool to investigate cancer metastasis.

## Introduction

Circulating tumor cells (CTCs) represent a biomarker for the detection, diagnosis, and prognosis of cancer, thereby serving as a liquid biopsy that may complement, or potentially replace, traditional tissue biopsies^1^. CTCs are shed into the bloodstream from both primary tumors and metastatic lesions, and they migrate through blood vessels, lymph capillaries, and bone marrow niches to reach distant sites, adhere to the inner wall of the blood vessels, and invade surrounding tissues^2^. In circulation, they are continuously exposed to mechanical stimuli such as compressive forces and shear stresses, and in response, they develop adaptive elastic and adhesive characteristics^3^. For example, CTCs reduce their stiffness (i.e., elasticity) and increase their deformability to overcome the diverse conditions of blood vessels and capillaries that could otherwise affect their invasive and metastatic activities^4^. Likewise, CTCs reduce their adhesiveness to the primary tumor to be able to escape it, and they increase their adhesiveness again once they reach the target host cell surface so that they can adhere to it^5^. Thus investigating cellular mechanics (i.e., elasticity and adhesiveness) of CTCs at the nanoscale offers great potential for identifying a metastatic biomarker and paves the way for new approaches in cancer diagnosis, prognosis, and clinical therapeutics^6^. However, the limited presence of CTCs in blood (~1–10 CTCs per mL of blood) and their inherent fragility make isolating these cells very challenging^7^. Although the malignant phenotype and metastatic potential of cancer cells have been shown to be correlated with their elastic properties^8^, the cellular mechanics of CTCs remain largely unexplored. Therefore, developing platforms that can efficiently isolate and nanomechanically characterize viable CTCs of varying phenotypes is a constantly evolving endeavor.

Microfluidic technology applies a number of different separation strategies for isolating CTCs from blood samples of cancer patients by exploiting differences in their affinity, physical, or electrical properties that distinguish them from other blood cells^9^. The ultimate goal is to capture CTCs with high purity (low background) and specificity, test their susceptibility to mechanical and chemical stimuli using approaches such as electrochemotherapy, and analyze their genome content. However, with these strategies, isolated cells are generally inaccessible externally for their nanomechanical characterizations. Ideally, intact CTCs need to be isolated in predefined confined areas such as microchannels or microwells, where they can be subsequently used in further quantitative nanomechanical characterizations.

Atomic force microscopy (AFM) is an ideal technique to quantitatively investigate the elastic and adhesive properties of cancer cells at the nanoscale^10^ due to its capability to measure tip–cell interactions under physiological conditions and with little damage caused to the cells. In a typical AFM force–distance measurement, the tip is brought toward and retracted from the cell surface; its interaction forces are monitored as a function of the cantilever’s displacement, and they are recorded as force–displacement curves. As a result, a wealth of quantitative information can be extracted from these curves in regard to cell elasticity and adhesiveness^11^.

Here we developed a combined microfluidic–AFM platform that is suitable for specific antibody-based capture of CTCs in whole-blood samples from prostate cancer patients and subsequent characterization of their elasticity and adhesiveness. The microfluidic device assembly occurs via reversible physical polydimethylsiloxane (PDMS)-to-glass bonding, which later allows external access to captured CTCs. Antibody activity on the platform is achieved through refined and robust chemistry that provides a high density of oriented (active) antibodies on the glass surface. By running simultaneous experiments, we show that the developed microfluidic device is highly efficient in capturing prostate CTCs via the epithelial cell adhesion molecule (EpCAM), the prostate-specific antigen (PSA), and the prostate-specific membrane antigen (PSMA). We also show that, when coupled with an AFM, the device enables the characterization of the elasticity and adhesiveness of captured intact CTCs residing on the glass substrate.

## Results and discussion

### Platform concept

**Fig. 1:**
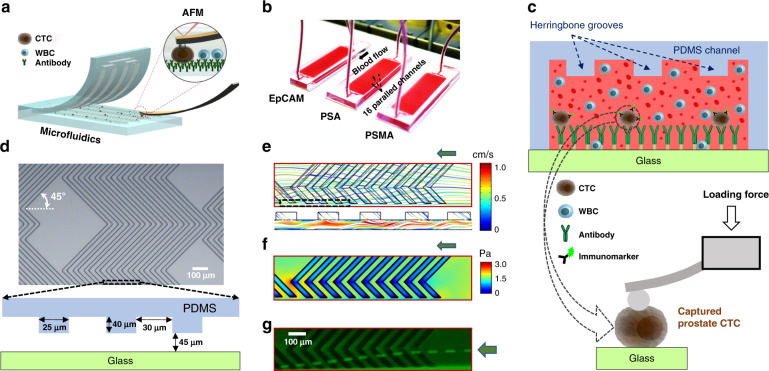
The microfluidic–AFM platform for the recognition and mechanophenotyping of CTCs from whole-blood samples of prostate cancer patients. **a** Scientific illustration of the developed platform. **b** A micrograph of the experimental set-up associated with the capture of CTCs from prostate cancer patients’ whole-blood samples. During the capture experiments, reversible physical PDMS-to-glass bonding was firm to prevent leakage of the blood. **c** The microfluidic device (top panel) is comprised of two reversibly (physically) bonded parts: a PDMS channel (16 total) integrated with HB elements and a glass substrate functionalized with capture antibodies (green Y shapes). Once captured, antigen-specific immunomarkers were used for the identification and enumeration of CTCs. The AFM tip engaging with a single captured intact prostate CTC is shown in the cartoon of the bottom panel, and measurements were performed after peeling off the PDMS chip from the glass slide. Schematics are not to scale. **d** A micrograph of the PDMS chip shows the geometry, periodicity, and size of the HB elements. **e** The simulated flow profile within the channel indicates a high degree of mixing at a 20 μL/min flow rate due to microvortices generated by the staggered HB elements. The green arrow shows the direction of the flow. The bottom panel shows 3D velocity streamlines, which indicates efficient mixing within the channel. **f** The estimated shear stress profile within the channel is shown. **g** The stacked time-lapse image of fluorescently labeled PC3 cells (bright green) spiked into blood visually confirms the simulated flow patterns

A scientific illustration of the developed microfluidic–AFM platform is shown in Fig. 1a, and its conceptual operation is schematically depicted in Fig. 1b. It consists of two reversibly (physically) bonded parts: a top array of PDMS channels incorporated with herringbone (HB) elements on their upper surfaces and a bottom glass substrate functionalized with capture antibodies (Fig. 1c). The channels (each 900 μm wide, 85 μm deep, and 48 mm long) are connected via single inlet and outlet ports; the HB elements (each 25 μm wide and 40 μm deep) are oriented at 45° with respect to longitudinal axes of the channels, and there are 30 μm gaps in between them (Fig. 1d). The HB elements create chaotic flow, which brings CTCs close to the glass surface so that their capture through antibody–antigen interactions is feasible^12^. The reversible physical bonding easily peels off of the PDMS chip for further AFM analysis of the captured intact CTCs residing on the glass substrate. While HB-based microfluidic devices have been widely used to isolate CTCs from blood samples of patients with epithelial (such as prostate^12^ and breast^13^) as well as nonepithelial (such as bone marrow^14^ and lymphocytic leukemia^15^) cancers, reversible microfluidic device assembly—which allows direct measurements of AFM tip–cell surface interactions with no restrictions on the external access of captured CTCs—to the best of our knowledge is novel.

### Visualization of the flow and shear

To examine the micromixing effect of staggered HB elements and the experienced shear stresses within the channels, we first visualized the flow and shear profiles using steady-state fluid dynamics simulation at a 20 µL/min flow rate (Supplementary Fig. S1). The results revealed that the HB elements effectively induce micromixing by generating microvortices (Fig. 1e). In addition, the estimated average shear stresses within channels were <2 Pa (Fig. 1f), which are expected not to jeopardize the captured cells remaining intact within the channels^16^. Finally, micromixing as well as cell intactness were experimentally verified by the flow patterns of cells spiked into blood samples (Fig. 1g).

### Microfluidic device assembly

**Fig. 2:**
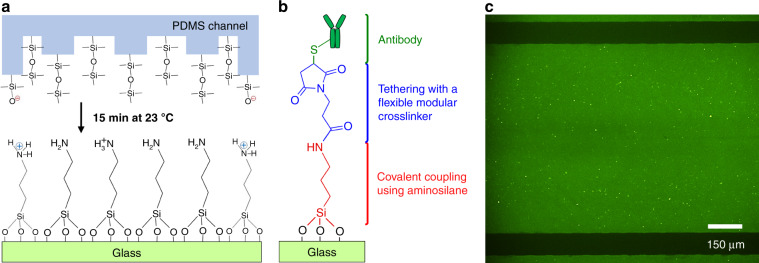
Reversible physical bonding of the PDMS microfluidic chip and APTES-silanized glass substrate. **a** The mechanism of the bonding occurs through silicon alkoxides (Si–O^−^) and protonated nitrogens (–N${{\mathrm{H}}_3^ +}$) at room temperature and under atmospheric pressure. This creates firm PDMS-to-glass attachment via NH–O hydrogen bonds. **b** After microfluidic device assembly, linker chemistry steps involving the reaction of amine (–NH_2_) groups with a modular crosslinker BMPS were used to covalently immobilize antibodies on the device glass surface. **c** The fluorescence micrograph of the green area within the microfluidic channel indicates that anti-EpCAM antibodies have been successfully immobilized with high density and the desired orientation (activity). The accessibility of the antigen-binding sites of anti-EpCAM antibodies was evaluated by labeling them with NHS-fluorescein

Plasma-activated permanent covalent bonding is a typical approach used in assembling microfluidic devices. Conversely, to enable reversible physical bonding in our study, we brought cleaned PDMS chips and (3-Aminopropyl)triethoxysilane (APTES)-silanized glass slides into contact at room temperature and under atmospheric pressure (Fig. 2a). Here the choice of APTES as a silanization strategy was unique in terms of generating a monolayer of densely packed and uniformly distributed (~1:1 ratio) protonated nitrogen (–N${{\mathrm{H}}_3^+}$) and reactive amine (–NH_2_) groups on the glass surface^17^ (Fig. 2a). In this way, the reversible physical bond between the PDMS and glass surfaces was feasible via NH–O hydrogen bonds formed between the silicon alkoxides (Si–O^−^)^18^ and protonated nitrogens (Fig. 2a).

Despite its simplicity, the use of APTES in the microfluidic reversible device assembly created firm, high-performance microfluidic channels that could withstand up to 150 μL/min flow rates with no leakage of blood, and they allowed further chemical modifications for the covalent immobilization of antibodies onto the glass substrate of the microfluidic device (Fig. 2b). Various chemistries have been reported for the isolation of cancer cells in microfluidic devices through antibody–antigen interactions^19^. Among them, avidin–biotin chemistry^20^ is widely used for strongly bonding antibodies to a glass surface via affinity-based linkages. However, in this chemistry, a solvent (ethanol) is used at each step of chemical modifications^12^. Therefore, covalent PDMS-to-glass bonding is required in the avidin–biotin approach to prevent leakages that could result from interactions between ethanol and PDMS (especially when working with small channel sizes). This bonding, however, makes device disassembly impossible, making isolated cells inaccessible and their external physical access (e.g., using AFM) is hindered.

### Glass activation and surface characterization

In addition to introducing firm reversible physical bonds between the PDMS chip and the glass slide, the deposition of APTES molecules on the glass substrate of the microfluidic device was also favored owing to the provision of reactive amine groups (Fig. 2a). As such, under proper conditions, these groups can interact with flexible tethered molecules so that antibodies are covalently attached to silanized glass surfaces in an oriented fashion (i.e., with antigen-binding arms pointing toward the glass surface). Therefore, we further applied a refined and robust antibody immobilization strategy by utilizing heterobifunctional *N*-[β-maleimidopropyloxy]-succinimide ester (BMPS) molecules as a modular crosslinker (Fig. 2b). Here each of the two reactive groups (succinimide and maleimide) within the molecule are designed for site-specific coupling: the succinimide was designed to couple with the amine groups of silane molecules through amide (N–C=O) linkages and the maleimide group was designed to couple with the sulfhydryl groups of antibodies (present in the lower portion of their Fc region)^21^ through thiol (C–S) linkages (Fig. 2b). Such site-specific immobilization of antibodies maximizes the accessibility of their two Fab domains (ligands)^22^ for binding to cell transmembrane antigens (receptors), such as EpCAM and PSMA. In addition, the rigidity of BMPS (spacer-arm length of 5.9 Å) offers high stability conditions for the antibodies when incubated in buffer solutions for prolonged times^17^.

**Fig. 3:**
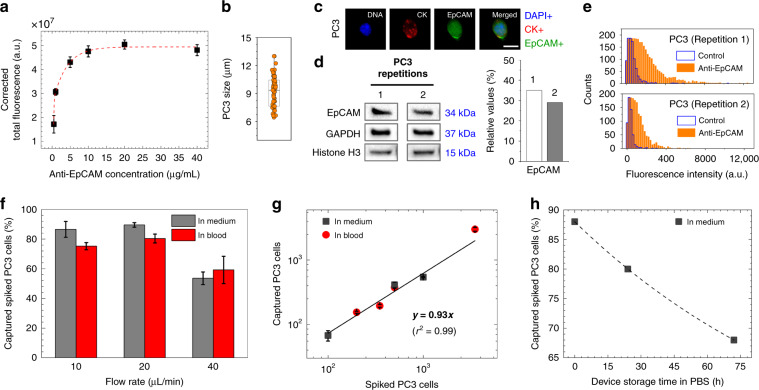
Device and cell characterization. **a** Variations in anti-EpCAM antibody adsorption capacity (*n* = 3) suggested that 10 μg/mL antibody concentration saturates maleimide-activated glass substrate (after BMPS deposition) of the microfluidic device. **b** Sizes of PC3 cells ranged from ~6 to 13 μm in diameter with an average size of ~9 μm. **c** Fluorescent image of a PC3 cell (DAPI+/CK+/EpCAM+), showing a cytoplasmic-to-nuclear area ratio of ~2.32. The scale bar is 10 μm. **d** Surface EpCAM protein levels in pelleted PC3 cells, as revealed by western blotting analysis, were ~32% overexpressed compared to the combined levels of GAPDH (control) and histone H3 (control). **e** Surface EpCAM protein levels in PC3 cells, as revealed by flow cytometry, verified that >75% of cell surface EpCAM binds to fluorophore-conjugated anti-EpCAM antibodies. **f** One milliliter of culture medium or blood sample was used to evaluate the capture of 1000 spiked in PC3 cells at 10, 20, and 40 μL/min flow rates. Among the tested flow rates, a 20 μL/min flow rate resulted in capture of ~90% of cells when spiked into culture medium (*n* = 4) and ~80% of cells when spiked into healthy blood (*n* = 3). **g** The spiked number of PC3 cells in culture medium (1000, 500, and 100 cells/mL, *n* = 3) and blood (3500, 500, 350, and 200 cells/mL, *n* = 3) showed good linear correlation with the captured number of cells. **h** Storing anti-EpCAM antibody-activated microfluidic devices for 3 days in PBS at 4 °C resulted in an ~20% reduction in the capture efficiency of PC3 cells spiked in culture medium. Error bars represent the mean ± S.D

The evaluation of the accessibility of the antigen-binding sites of antibodies (e.g., using anti-EpCAM) was carried out by labeling them with 5/6-carboxyfluorescein (green) succinimidyl ester (NHS-fluorescein). A characteristic of this molecule is that its succinimide groups bind specifically with the N-terminal groups of antibodies that are located at the ends of their Fab arms^21^. In this way, we were able to show that anti-EpCAM antibodies have been reliably immobilized on glass surfaces with high density and the desired orientation (Fig. 2c). In addition, to investigate the amount of saturated antibody adsorption, anti-EpCAM antibodies were diluted at concentrations of 0.5, 5, 10, 20, and 40 μg/mL in phosphate-buffered saline (PBS) and incubated on the BMPS-activated glass substrates (after APTES silanization) of microfluidic devices. The change in NHS-fluorescein intensity as measured from images (Supplementary Fig. S2) was used to track the surface concentrations of deposited antibodies. The results revealed that a 10 μg/mL antibody concentration was enough to saturate the capacity of maleimide substituents on the device glass surfaces (Fig. 3a). Hence, 10 μg/mL was selected as the optimal concentration for effective and reproducible cancer cell isolation results.

The antibody-loading capacity of maleimide-activated glass substrates, shown in Fig. 3a, is independent of the choice of antibodies due to highly specific site-specific coupling between maleimide groups and the sulfhydryl groups of antibodies. Thus, when the amount and orientation of anti-PSA and anti-PSMA antibodies on the glass substrates is unknown, we predict^17^ that these antibodies will saturate maleimide-activated glass substrate in the same manner as anti-EpCAM antibodies did. Characterization involving immobilization of these antibodies, however, is left for future studies.

Finally, to investigate the effects of surface chemical compositions on the anti-EpCAM antibody immobilization efficacy, several experiments were conducted using cleaned, APTES-silanized, and BMPS-activated glass substrates of the microfluidic devices (Supplementary Fig. S3a). As expected, the NHS-fluorescein signal of the anti-EpCAM antibodies covalently linked to APTES was ~2.5 times less intense than it was when they were anchored using BMPS. This finding further confirmed that BMPS is needed for high-density immobilization of antibodies onto glass substrates of the microfluidic device.

### Cell characterization, cell capture optimization, and antibody functional stability

To ensure reproducibility in cell capture optimization experiments and obtain good correlation of the results with the capture of CTCs, we first investigated the size and EpCAM expression levels of human prostate PC3 cells. Overall, our results revealed that the average size of PC3 cells was 9.3 ± 1.7 μm in diameter (Fig. 3b) and that they showed a differential response to staining of their nuclei (DNA), cytoplasmic cytokeratin (CK), and surface EpCAM (Fig. 3c). By western blotting analysis, we further verified that surface EpCAM protein levels in pelleted PC3 cells (~10^6^ cells/mL concentration) were 32% ± 4% more highly expressed than the control glyceraldehyde-3-phosphate dehydrogenase (GAPDH) and histone H3 combined (Fig. 3d). In connection, flow cytometry revealed that, in >75% of PC3 cells (~10^6^ cells/mL concentration), surface EpCAM proteins were positively immunostained using fluorophore-conjugated anti-EpCAM antibodies (Fig. 3e). With these results, and after functionalizing microfluidic devices with antibodies, we proceeded with the cell capture optimization experiments using PC3 cells spiked into 1 mL of either culture medium or whole healthy blood samples. To this end, anti-EpCAM antibodies were immobilized on the devices at a concentration of 10 μg/mL, and cells were labeled with green fluorescent dyes to monitor the cell capture dynamics. Cell capture characterizations were based on varying flow rates (10, 20, and 40 μL/min), cell concentrations (10, 100, 200, 500, 1000, and 3500 cells/mL), and device storage times (0, 24, and 72 h). The cell capture efficiency was evaluated as the fraction of captured cells with respect to the initial number of cells loaded into the device. Altogether, our results showed that a 20 μL/min flow rate resulted in a capture of 89.5% ± 1.5% of the PC3 cells when spiked in culture medium at 1000 cells/mL concentrations (Fig. 3f). Testing the capture efficiencies of PC3 cells at the individual steps involved in the linker chemistry, with anti-EpCAM antibodies at 10 μg/mL concentrations or without (control), further verified that high cell capture efficiency requires immobilization of antibodies via APTES-BMPS chemistry (Supplementary Fig. S3b). For example, at 20 μL/min flow rates, ~42% PC3 cell capture was achieved with antibodies immobilized through APTES alone, while only ~6% capture efficiency was achieved when antibodies were physically adsorbed on cleaned glass surfaces. When no antibodies were used, these values dropped to ~21% and ~1%, respectively. Meanwhile, a previously reported HB microfluidic chip (HB-Chip^12^) used anti-EpCAM antibodies immobilized (with avidin–biotin chemistry) on all of the internal channel walls, which resulted in ~92% PC3 cell capture efficiency. In comparison, the slightly lower capture efficiency (~90%) in our device was due to the low cell capture events on untreated PDMS channel walls (through physically adsorbed antibodies), which contributed only ~7% to the total cell capture efficiency (Supplementary Fig. S4). However, in our study, it was important that cells were mostly captured on glass surfaces for downstream AFM analysis.

Variations in flow rates showed a minimal impact on PC3 cell capture efficiency (e.g., 86.6% ± 5.3% cell capture at 10 μL/min flow rate) until the flow rate was increased to 40 μL/min, which resulted in an ~35% reduction in cell capture percentage (Fig. 3f). Similar results were observed when 1000 PC3 cells were spiked into 1 mL of healthy donor blood samples, with 75.2% ± 2.4%, 80.4% ± 2.9%, and 59.2% ± 9.2% cell capture efficiencies at 10, 20, and 40 μL/min flow rates, respectively (Fig. 3f). In agreement, regression analysis demonstrated that, in both culture medium and blood samples, the microfluidic device showed equal responses (linear slope of 0.93, *r*^2^ = 0.99) to cell capture when ≥100 cells were spiked into samples, and there was good reproducibility (Fig. 3g). However, when 10 cells were spiked into 1 mL of culture medium or blood samples, the recovery rates dropped to 20–30% (*n* = 3), likely due to pipetting errors and uncertainties in the initial number of spiked cells. In addition, cell settling within the syringes and tubes was another contributing factor. Once captured, no cells were observed to dislocate or detach upon facing varying flow rates due to specific capture of cells by the anti-EpCAM antibodies. Moreover, live/dead staining verified that there was no cell death.

Another set of experiments was performed to investigate the overtime (e.g., days) functional stability (when stored in PBS at 4 °C) of anti-EpCAM antibody-activated devices for 0, 24, and 72 h. Figure 3h shows the result of the captured PC3 cell counts, spiked in culture medium, as a function of device storage times. The cell capture rate was slightly decreased (from 88% to 68%), suggesting that microfluidic devices coated with antibodies can still be effective in capturing cancer cells when stored for prolonged times (e.g., >3 days).

Finally, given that anti-EpCAM is the most widely used antibody in affinity-based capture of CTCs^23^ and that the PC3 cell line is well characterized in terms of the size and surface antigen expression (EpCAM+/PSA−/PSMA−) of the cells^24^, we used PC3 cells to optimize the device capture efficiency using anti-EpCAM antibodies and obtained sensitivity data. Future studies will include other cell lines expressing PSA and PSMA (e.g., LNCaP and DU145) as models in additional device characterization experiments.

### Isolation of prostate CTCs from whole-blood samples of cancer patients

Next, we tested the applicability of the developed microfluidic device in capturing prostate CTCs from blood samples of patients with cancer at different stages. In this context, we collected blood samples from six prostate cancer patients at the Mediclinic City Hospital in Dubai, UAE. The profiles of the patients are summarized in Table 1. Briefly, at the time of collection, 4 of the patients (Patients 1, 2, 3, and 4) were under active surveillance (i.e., with PSA levels <10 ng/mL and no identified metastatic sites), 1 patient (Patient 5) was diagnosed with bone metastasis (PSA level >50 ng/mL), and 1 patient (Patient 6) was diagnosed with lymph node metastasis (PSA level >100 ng/mL).

**Table 1:** Isolation of CTCs from whole blood samples of prostate cancer patients^a^

Patient^b^	Age	Gleason score	PSA (ng/mL)	Blood (mL)	CTC capture		
					Anti-EpCAM	Anti-PSA	Anti-PSMA
1	65	6 (3 + 3)	<10	3	5	6	5
2	66	6 (3 + 3)	4.8	3	9	2	5
3	61	6 (3 + 3)	7.0	3	9	2	6
4	64	7 (3 + 4)	0.1	1	3	—	—
5	64	7 (3 + 4)	>50	1	14	—	—
6	72	10 (5 + 5)	>100	1	17	—	—

— no data available

^a^All experiments were conducted using whole-patient blood at a 20 μL/min flow rate and 10 μg/mL antibody concentrations

^b^The metastatic sites for patients 1, 2, 3, 4, 5, and 6 were none, none, none, none, bone, and lymph nodes, respectively

Studies have suggested that the level of antigen expression on CTCs dynamically changes during their circulation in the bloodstream, with some cells downregulating their antigen expression during metastasis. For example, it was shown that surface expression of EpCAM on CTCs might be more heterogeneous than initially expected due to their invasive and metastatic behavior^25,26^, and other work showed that heterogeneous PSMA expression level on CTCs is related to genetic mutations^27,28^. Thus, to enhance the capture of CTCs from patients with localized cancer, we integrated three different antibodies, namely, anti-EpCAM, anti-PSA, and anti-PSMA. Each of the whole-blood samples (3 mL) was then passed simultaneously and under identical conditions through the microfluidic devices, as shown in Fig. 1b. For metastatic cancer patients, on the other hand, whole-blood samples (1 mL) were passed only through anti-EpCAM chips due to the limited volume of blood we were able to obtain (i.e., only 1 mL).

**Fig. 4:**
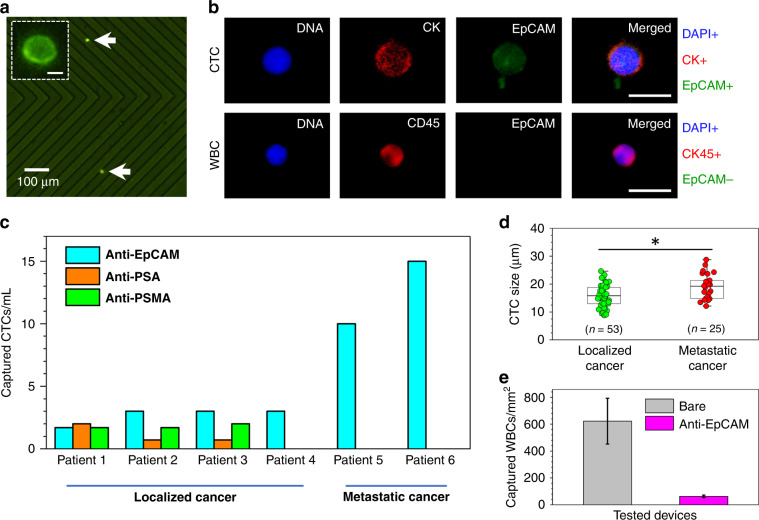
Isolation of CTCs from patients with localized and metastatic prostate cancer. **a** Example CTCs (bright green dots) captured from whole blood of localized cancer patients using an anti-EpCAM antibody-activated microfluidic device. A magnified single captured intact CTC is shown in the inset. The scale bar is 8 μm. **b** Fluorescent images of a captured CTC (top panel) and a WBC (bottom panel) revealed the staining of their nuclei (blue, DAPI+ for CTC and WBC), cytoplasmic cytokeratin (red, CK+ for CTC), CD45 (red, CD45+ for WBC), and EpCAM (green, EpCAM+ for CTC and EpCAM− for WBC). The cytoplasmic-to-nuclear area ratio of the CTCs was ~1.6. The scale bar is 20 μm. **c** The developed device has shown to be efficient in capturing CTCs both from patients with localized (using anti-EpCAM, anti-PSA, and anti-PSMA antibodies in Patients 1–3 and using anti-EpCAM in Patient 4) and metastatic prostate cancer (using anti-EpCAM antibodies in Patients 5 and 6). **d** The size distribution of captured CTCs revealed that, on average, the size of CTCs from localized cancer is ~16 μm in diameter, whereas the size of CTCs from metastatic cancer is ~19 μm in diameter. *Statistically not significant at *P* < 0.05 using *t* test. **e** Nonspecific binding of WBCs in EpCAM antibody-activated microfluidic devices was ~10 times less than that of the bare (untreated) chips due to hydrophilicity of the high-density antibody layer on the glass substrates (*n* = 6). Error bars represent the mean ± S.D

After the capture experiments, anti-EpCAM antibodies conjugated to green fluorophores were used to stain captured CTCs, which helped us in their identification and enumeration under a fluorescence microscope (Fig. 4a). To ensure that captured EpCAM+ cells were CTCs, additional immunostaining was applied to EpCAM+ cells to confirm the presence of their DNA and CK^12^. The results revealed that the captured EpCAM+ cells in our study were CTCs, as they were positive for nuclear (DNA+) and cytoplasmic CK (CK+) staining (top panel in Fig. 4b). As expected^12,29^, white blood cells (WBCs) in the background did not show a positive response to immunostaining of EpCAM, but they were positive for the transmembrane marker CD45 (bottom panel in Fig. 4b). Additional work in verification of the CD45+/EpCAM− characteristics included immunostaining and flow cytometry of WBCs obtained through Ficoll density gradient separation (Supplementary Fig. S5). The results verified that WBCs do not bind to anti-EpCAM, anti-PSA, and anti-PSMA but only to anti-CD45 antibodies, confirming their CD45+/EpCAM−/PSA−/PSMA− characteristics.

In all experiments, using 10 μg/mL antibody concentrations (regardless of the antigen choice) and 20 μL/min flow rates provided successful capture of CTCs from the patient samples (Fig. 4c and Table 1). As such, when combined, the average captures were 2.2 ± 1.0 CTCs/mL, 1.1 ± 0.8 CTCs/mL, and 1.8 ± 0.1 CTCs/mL using anti-EpCAM, anti-PSA, and anti-PSMA antibodies, respectively (from patients with localized cancer), and 12.5 ± 3.5 CTCs/mL using anti-EpCAM antibodies (from patients with metastatic cancer). Obviously, the transmembrane EpCAM and PSMA represent an ideal route for the specific antibody-based capture of CTCs, as their expression levels have been shown to increase with cancer progression^30,31^. The capture of CTCs through PSA, however, is interesting and needs careful interpretation, since these proteins are known to be only secreted by CTCs but are not present on their surfaces^32^. Therefore, we assume that CTC capture occurred through PSAs during their secretion. Nevertheless, the potential disadvantage of targeting PSA on CTCs could result from the total serum PSA in blood, which may compete to bind available antigen-binding sites of the anti-PSA antibodies, thus lowering the capture efficiency of CTCs. As predicted^33^, due to the monoclonal nature of the anti-EpCAM, anti-PSA, and anti-PSMA antibodies used in this study, their affinity for capturing EpCAM+, PSA+, and PSMA+ CTCs was specific.

Finally, analyzing fluorescent images of captured CTCs revealed that the average size of CTCs from localized cancer samples was 15.7 ± 4.1 μm, whereas the average size of CTCs from metastatic samples was 19.1 ± 4.1 μm (Fig. 4d), but the difference between the two groups was not significant. Compared to the average size of WBCs (~6 to 10 μm) and PC3 cells (~6 to 13 μm), these CTCs were at least 1.2 times larger.

Generally, it is expected that prostate CTCs are much more rare in the blood samples of patients with localized cancer than they are in samples from patients with metastatic cancer^34^. For example, in a clinical study involving 120 prostate cancer patients^35^, the median CTC count for patients with no primary treatment was 2.5 per 7.5 mL of blood, whereas for patients with bone metastasis, the median CTC count was 10.5 cells per 7.5 mL of blood. In the work conducted with the HB-Chip^12^, depending on the disease state, the number of captured CTCs from 17 patients with metastatic prostate cancer varied between 12 and 3167 per mL of blood. It is also expected that the cell size of CTCs could vary from patient to patient and depend on the status of the cancer. For example, one study^36^ reported that CTCs isolated from 16 prostate cancer patients have an average size of 7.97 ± 1.81 μm. Another study^37^ alternatively reported that CTCs isolated from 18 prostate cancer patients have an average size of 15.9 ± 6.9 μm. Consistent with our results, it appears that the variation in the counts and size of captured prostate CTCs is an expected outcome in relation to the patients’ clinical status. Therefore, analysis of the number and sizes of captured CTCs potentially holds promise as a diagnostic and prognostic tool for prostate cancer; however, further studies are still required to identify whether the differences are statistically significant.

Finally, the results with anti-EpCAM antibody-activated devices showed that there were an average of ten times fewer nonspecifically bound WBCs per mm^2^ of channel area compared to devices with only cleaned (non-active) glass substrates (Fig. 4e and Supplementary Fig. S6). Here we hypothesize that the hydrophilicity^38^ of high-density, oriented antibodies (Fig. 2c) immobilized on glass surfaces provided natural surface prevention of the capture of WBCs. In addition, analyzing fluorescent images of WBCs showed no false positive cases of DAPI+/CK+/EpCAM+staining. Finally, when antibody-based isolation of CTCs is used, depending on the choice of chemistry, the capture surfaces are often subject to high levels of cross-reactivity with off-target cells (e.g., erythrocytes and leukocytes)^39^. For example, in our previous study^40^, we observed a background of erythrocytes and leukocytes when avidin–biotin chemistry was used to immobilize antibodies in the microfluidic device, where avidin’s strong positive charge^41^ most likely caused ionic interactions with these cells. Likewise, using avidin–biotin chemistry, an HB-Chip^12^ reported 14% purity of captured spiked in PC3 cells among captured WBCs in the channels, while in our device the capture purity of PC3 cells spiked into blood was twice as high as that result (28.0% ± 3.6%). Nevertheless, when the purity of captured CTCs with reference to WBCs was studied in our chip, a significant decrease was observed (8.7% ± 3.9%), which was perhaps due to lower EpCAM expression on CTCs going through the epithelial-to-mesenchymal transition^2,4^.

### AFM-based characterization of cancer cells

**Fig. 5:**
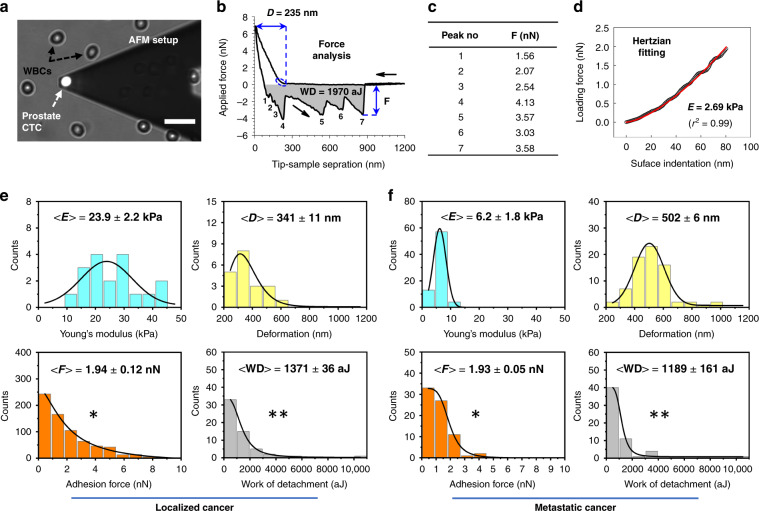
AFM analysis of the microfluidic-captured intact prostate CTCs of patients with localized (Patient 4) and metastatic (Patient 6) cancer. **a** A micrograph shows an AFM tip positioned above the center of EpCAM-stained CTC so that force measurements could be performed for follow-up nanomechanical characterization. The scale bar is 30 μm. **b** A representative force curve demonstrates the interaction between the AFM tip and the CTC, where black arrows represent the tip’s approach to and retraction from the cell surface. Following the initial tip–cell surface contact, the cell experiences an amount of surface deformation “*D*” due to the applied constant loading force. When the tip is retracted from the cell surface, the cell experiences a number of adhesion forces “*F*” (peaks 1–7 in the retraction process) due to its interactions with the AFM tip surface until the tip is completely detached from it. In the process, the work of detachment “WD” (shaded gray area below the zero-force line) indicates the amount of work needed to completely detach the tip from the cell surface. **c** The measured adhesion values are tabulated for all peaks in **b**. **d** After the initial tip–cell surface contact, force data (black) were fit (red) to surface indentation (~80 nm, dashed blue ellipse in **b**) to estimate the local Young’s modulus “*E*”. **e**, **f** Histograms of the distribution of all Young’s moduli *E*, deformation *D*, adhesion forces *F*, and work of detachment WD, as measured for CTCs of localized (*n* = 3) and metastatic (*n* = 2) cancer origins. Solid black lines are data fit to lognormal or Gauss probability density functions. Data represent the mean ± S.D. *Statistically significant and **statistically not significant at *P* < 0.05, as assessed by *t* tests

In our study, AFM was utilized to collect proof-of-principle elastic and adhesive measurements of intact prostate CTCs—captured from the whole-blood samples from localized and metastatic cancer patients (Patient 4 and Patient 6, respectively). After the disassembly (peeling off) of the PDMS chip from the glass slide, immunostained captured intact cancer cells residing on anti-EpCAM-activated glass substrates were transferred onto an AFM stage for high-resolution force measurements (Fig. 5a). Here the specific antibody–antigen interaction allowed cells to remain firmly attached to the glass substrate during the disassembly of the device, and the highly hydrophobic nature of the APTES-silanized glass substrate (through C–Si alkyl substituents^42^) provided a protective shield for the captured cells, so the majority of cells (>60%) remained intact (Supplementary Fig. S7). After the measurements, the data collected from AFM force curves were quantified (Fig. 5b–d) for Young’s modulus (elasticity), deformation, adhesion forces, and work of detachment. In sum, histograms revealed that CTCs of localized cancer origin are more stiff (23.9 ± 2.2 kPa elasticity and 341 ± 11 nm deformability) than CTCs of metastatic cancer origin (6.2 ± 1.8 kPa elasticity and 502 ± 6 nm deformability); data are shown in the top panels of Fig. 5e, f. In contrast, no difference between the groups of CTCs was observed when their adhesion to the AFM tip surface was analyzed (bottom panels in Fig. 5e, f). However, as reflected in histogram broadening of CTCs with localized cancer background, the difference was significant in terms of multiple adhesion events observed in both groups, suggesting that more surface adhesins are expressed on the surfaces of CTCs from localized cancer than are found on those from metastatic cancer. This finding also verified that the average work of detachment was slightly lower in metastatic CTCs than in their counterparts (bottom panels in Fig. 5e, f).

CTCs in the metastatic tumor are hypothesized to develop decreased elasticity and adhesiveness and increased deformability as a part of epithelial-to-mesenchymal transition^2,4^; these changes allow the cells to detach and traverse through diverse microenvironments of blood vessels, lymph capillaries, and bone marrow niches. To utilize the mechanical properties of cancer cells as biomarkers to distinguish cancer cells from benign cells, one study^43^ used AFM to identify mechanical property changes in live metastatic cancer cells extracted from the body fluids of lung, breast, and pancreatic cancer patients. The results showed that metastatic cancer cells exhibit lower elasticity (higher deformability) and adhesiveness than normal cells. More importantly, in another study^44^, AFM showed that in the analysis of >100 metastatic prostate CTCs (isolated through porous membranes), depending on the cancer progression, the elasticity, the adhesion forces, and the deformations of CTCs could range from 0.29 to 8.60 kPa, from 0.05 to 1.15 nN, and from 101 to 1287 nm, respectively. Taken together, the decrease in the elasticity and the increase in the deformability of metastatic CTCs in our work were anticipated results, and their values seemed to be within the reported values of the mechanical properties of clinical metastatic prostate CTCs.

Although our AFM measurements were based only on 5 CTC samples (3 with localized cancer background, Patient 4; and 2 with metastatic cancer background, Patient 6), the AFM tip probed at least 64 points on the surfaces of each of these cells. Thus we believe that our AFM results are important in terms of providing insights into the mechanical differences between CTCs from cancer at different stages. Nevertheless, our future work will consider a higher number of CTCs for statistical significance in AFM measurements. For example, cells from several patients with prostate cancer at different stages, patients undergoing different therapies, and patients with different therapy outcomes will be taken into account. In this way, the nanomechanical properties of CTCs could be correlated to the disease status and could be investigated as biomarkers, which are potentially also correlated with drug response and resistance.

It is worth noting that while the filtration methodology reported in ref. ^44^ was effective in isolating a large number of CTCs from patients with metastatic prostate cancer, its efficiency is limited by fixed pore sizes, which could technically challenge the isolation of CTCs with smaller diameters. Clogging and a high degree of contamination with WBCs^45^ are additional disadvantages to be expected. The microfluidic device reported in this study, on the other hand, overcomes these challenges and is reproducible and straightforward in its ability to isolate cancer cells and characterize them with AFM.

Finally, additional AFM measurements were conducted with PC3 cells to compare their mechanical properties with those of CTCs. The results revealed that PC3 cells are more elastic (with 2.53 ± 0.13 kPa), more easily reshaped (with 1493.4 ± 567.1 nm), and more adhesive (with 3.79 ± 0.52 nN), even when the applied constant loading forces were at least ~2 times less than those applied to the CTCs (Supplementary Fig. S8).

## Conclusions

We demonstrated successful integration of microfluidics with AFM for the antibody-based capture and subsequent nanomechanical characterization of CTCs from whole-blood samples of prostate cancer patients (both with localized and metastatic cancer). We applied refined and robust APTES-BMPS chemistry to covalently immobilize anti-EpCAM antibodies with high density and the desired orientation on the glass substrate of the microfluidic chip. Using prostate PC3 cells as a model cell line, we showed that the device is highly specific for and sensitive in capturing cells expressing surface EpCAM proteins. In addition, we demonstrated that the antibodies preserve their functional activity when the device is stored for prolonged times (e.g., days). We also developed a unique reversible physical bonding mechanism between the PDMS chip and the APTES-silanized glass substrate via NH–O hydrogen bonds, and we showed that, by simply peeling off the PDMS, the captured intact cancer cells residing on the glass substrates could be accessed externally for follow-up elasticity and adhesion measurements using AFM.

CTC capture results revealed that the functionalized microfluidic devices are highly efficient in isolating CTCs from patients with localized cancer using anti-EpCAM, anti-PSA, and anti-PSMA antibodies; anti-EpCAM exhibited the highest capture efficiency, and it was followed by anti-PSMA and anti-PSA. Interestingly, devices with the anti-PSA antibody showed variable efficiency in CTC capture across patients. However, this finding needs further investigation, since PSA proteins are known to be secreted by prostate cells but are not present on their surfaces. In comparison, an increasing trend was observed in the number of CTCs captured from patients with metastatic cancer using anti-EpCAM antibodies. Further investigation of the size of captured CTCs revealed that CTCs are on average smaller in localized cancer than they are in metastatic cancer. In addition, compared to the average size of WBCs (~6–10 μm) and PC3 cells (~6–13 μm), the size of CTCs was at least 1.2 times larger. Finally, AFM nanomechanical measurements showed that metastatic CTCs are softer and more easily reshaped than those from localized cancer. Although for both groups, the average adhesion of CTCs to the AFM tip surface remained the same, the presence of multiple adhesion events in metastatic CTCs was less extensive than it was in their counterparts, indicating lower expression of cell surface adhesins. Overall, these findings suggest that investigating the number, size, and nanomechanical properties of prostate CTCs could potentially open a door in identifying metastatic biomarkers. However, for statistical significance, future studies need to include a higher number of CTCs from patients with different stages of cancer.

In this work, our goal was to bring together microfluidic CTC capture and AFM nanomechanical characterization in a reliable and streamlined process. With our microfluidic device, we have reported the use of an alternative chemistry that immobilizes anti-EpCAM antibodies at a high density and with the desired orientation only on the glass substrate of a device, which allows disassembly of the device for coupling with AFM. The method developed here for recognizing and nanomechanically characterizing prostate CTCs is simple, robust, and reproducible. We anticipate that this strategy can be used to capture prostate CTCs with high accuracy, specificity and sensitivity, and mechanophenotype, and it can do so with high precision and minimally destructive repeatable measurements. The mechanophenotypic profiling of cancer cells at the single-cell level can potentially provide a promising solution for the study of cancer metastasis. The platform can also be adapted to other types of cancers by simply modifying the capture antibodies.

## Materials and methods

### Chip design and fabrication

The design of the PDMS chip was adapted from our previously developed device^40^. The chip fabrication was carried out on a silicon wafer with two layers of high-viscosity negative photoresist SU-8 (MicroChem Corp., USA) using a standard soft photolithography protocol: one for the microfluidic channels and the other for the HB elements. After development, a 10:1 (w/w) mixture of the base elastomer Sylgard 184 and a curing agent (Dow Corning Corp., USA) was poured onto the wafer to form a PDMS chip at a thickness of ~5 mm. The wafer was then placed in a vacuum desiccator for 1 h to remove the air bubbles generated within the PDMS during the mixing step. After curing in a 60 °C oven for 2 h, the PDMS chip was peeled off from the wafer, cut into ~75 mm × 25 mm pieces, and punched at its inlet and outlet ports. Each PDMS chip was then cleaned ultrasonically in deionized (DI) water and in ethanol for 3 min to remove large particles from the surfaces, then each chip was dried with nitrogen and stored under vacuum.

### Glass cleaning and silanization

Glass slides (75 mm × 25 mm, J. Melvin Freed Inc., USA) were ultrasonically cleaned in DI water and ethanol for 3 min, dried with nitrogen, and ultraviolet/ozone cleaned for 30 min. They were then soaked in an anhydrous toluene (>99.5%) solution containing 0.5% (wt) APTES (Sigma-Aldrich) molecules for 45 min. After silanization, the glass substrates were sonicated in ethanol for 3 min to remove loosely bound molecules, and then they were dried with nitrogen and placed in a 100 °C oven for 30 min to allow APTES annealing^17^.

### Microfluidic device assembly, antibody linking, and surface characterization

After the APTES annealing process, the cleaned PDMS chips were immediately brought into contact with APTES-silanized glass slides at room temperature and under atmospheric pressure to form reversibly (physically) bonded microfluidic devices. The devices were then kept under vacuum for 15 min to allow the trapped gas bubbles present between the surfaces to escape naturally.

The immobilization of anti-human monoclonal 9C4, YPSMA-1, and C-19 antibodies (Santa Cruz Biotechnology) against EpCAM, PSA, and PSMA antigens, respectively, was accomplished through a two-step^17^ channel pipetting process: in step one, a 3 mg/mL solution of BMPS (Fisher Scientific) was passed through PBS and incubated for 30 min; and in step two, a 10 μg/mL antibody concentration (diluted in PBS) was added and incubated for 45 min. After each step, PBS was used to flush the channels so that unbound molecules/antibodies were removed. Afterward, 1% (v/v) bovine serum albumin was incubated for 20 min to block the nonspecific binding sites on glass substrates. Then droplets of PBS were pipetted on the inlet and outlet ports of the devices to prevent any liquid evaporation within the channels.

The surface concentrations and orientations of the anti-EpCAM antibody-activated microfluidic devices were verified by adding a solution of 1 mg of NHS-fluorescein (excitation maximum 495 nm and emission maximum 519 nm, Fisher Scientific) in 400 μL of dimethylformamide (Sigma-Aldrich) in the dark and allowing incubation for 60 min. Meanwhile, control experiments were carried out with and without antibodies at each step of the linker chemistry reactions. After washing the channels with PBS to remove unbound dyes, the glass substrates were detached from the PDMS chips and immersed in Petri dishes containing dye-free PBS. Afterward, imaging was carried out with a Nikon Eclipse Ti inverted fluorescence microscope using a fluorescein isothiocyanate (FITC) filter cube and through a ×10 air objective. The corrected total fluorescence of images was calculated following an algorithm that scales and shifts each pixel value of the input image (control, 0.5 μg/mL anti-EpCAM antibody concentration) to match its mean and standard deviation to those of target images (corrected)^46^. Open-source frameworks G’MIC (https://gmic.eu) and ImageJ (https://imagej.nih.gov/ij) were used in processing the fluorescence images as follows: first, a G’MIC color transfer filter was used to “impose” (transfer) the color characteristics of one image to another. For each set, fluorescent images of experiments with 0.5 µg/mL antibody concentration were used as a control. Afterward, images were analyzed in ImageJ to calculate the corrected total fluorescence of images using the equation:

1 $${\mathrm{IntDen}}=\left( {{\mathrm{Area}}} \right) \times \left( {{\mathrm{MGVSample}}} \right),$$

where IntDen (integrated density) = a measure of “grayness” on an individual selection, Area = area of interest, MGVSample (mean gray value of the sample) = the sum of the gray values of all the pixels in the selection divided by the number of pixels. Using Eq. 1, corrected total fluorescence (CTF) was then calculated using the equation:

2 $${\rm{CTF}} = {\rm{IntDen}}\,-\,\left( {{\rm{Area}}\,\times\,{\rm{MGVBackground}}} \right)\,=\,\left( {{\rm{Area}}} \right)\,\times\,\left( {{\rm{MGVSample}}\,-\,{\rm{MGVBackground}}} \right).$$

### Cell culture

The PC3 human prostate cancer cell line (American Type Culture Collection) was used as a model cancer cell line in all device capture optimization experiments. Prior to each experiment, cells were grown in Roswell Park Memorial Institute (Sigma-Aldrich) culture medium complemented with 10% fetal bovine serum (Sigma-Aldrich) and 1% penicillin–streptomycin (Sigma-Aldrich) in a humidified incubator with 5% CO_2_ at 37 °C. After 3–4 passages (when cells were ~80% confluent), cells were washed three times with PBS and then were fluorescently labeled using CellTracker green dyes (excitation maximum 492 nm and emission maximum 517 nm, Fisher Scientific) for 15 min at room temperature in the dark. Next, they were trypsinized using TrypLE express enzyme (Sigma-Aldrich) and centrifuged at 900 rpm for 5 min. The cell pellets (~10^7^ cells) were then resuspended in fresh culture medium. A trypan blue exclusion assay (Sigma-Aldrich) was applied to directly count the number of live cells using a Countess II FL automated cell counter (Fisher Scientific).

### Western blotting and flow cytometry

Western blotting was used to determine the expression level of surface EpCAM proteins in pellet PC3 cells (~10^6^ cells/mL concentration). Radioimmunoprecipitation assay buffer with protease inhibitor cocktail (Roche) was used to lyse the cells. Protein concentrations from whole-cell lysate were measured using a Bradford Assay Kit (Fisher Scientific). Proteins (30 µg/mL) were loaded onto gels and were separated using sodium dodecyl sulfate–polyacrylamide gel electrophoresis. The separated proteins were transferred to polyvinylidene difluoride membranes by a wet transfer method and then were incubated with anti-EpCAM primary antibodies (1:500 dilution) and anti-mouse secondary antibodies (1:1000 dilution). Anti-Histone H3 antibodies (1:1000 dilution, Abcam) and anti-GAPDH antibodies (1:1000 dilution, Abcam) were used as loading controls. The immunoreactive bands were imaged using a ChemiDoc MP Imaging System (BioRad).

Flow cytometry was used to further analyze the expression of EpCAM in PC3 cells. PC3 cells (~10^6^ cells/mL concentration) were pelleted, and anti-EpCAM antibodies (5 µL dissolved in 95 µL PBS) conjugated to green fluorophores (FITC) were used to stain cells for 30 min at room temperature in the dark. After washing the cells twice, flow cytometric signals were measured using BD FACSAria III (BD Biosciences). Cell populations with no attached conjugated antibodies were used as controls.

### Blood collection

Blood samples from 4 healthy donors and from 6 patients with localized and advanced prostate cancer were collected into tubes containing ethylenediaminetetraacetic acid and processed in microfluidic devices within 3 h of blood draw; the process was performed as approved by the NYU Abu Dhabi Institutional Review Board.

### Cell capture optimizations

Fluorescently labeled (CellTracker green dyes) PC3 cells were spiked into 1 mL of culture medium/healthy donor blood samples at 1000 cells/mL concentrations. The flow rates were controlled with a neMESYS syringe pump (Cetoni GmbH, Germany). During the experiments, cells were contained in glass/plastic syringes and passed into the microfluidic channels using polyethylene tubes. Following capture, channels were washed with 1 mL of PBS at the flow rate used to remove any unattached cells. The capture efficiency of the device was obtained as follows: PC3 cells were first counted manually at the inlet while cells were entering the device and then within the device after cell capture. These numbers were then divided and recorded as the percentage of cell capture. Similarly, in another set of experiments, the regression analysis was carried out by spiking PC3 cells into 1 mL culture medium/healthy donor blood samples at different concentrations and passing through channels at 20 µL/min flow rates. The captured number of PC3 cells was counted and recorded on a logarithmic scale as a function of the spiked number of cells.

### CTC capture and background and purity evaluation

Prostate cancer patients’ blood samples (3 mL with localized cancer and 1 mL with metastatic cancer) were transferred to plastic syringes and passed over the antibody-activated microfluidic channels at 20 µL/min flow rates using a neMESYS syringe pump. After washing channels with 1 mL of PBS, the captured CTCs were immunostained with anti-human monoclonal C-10 (anti-EpCAM) antibodies conjugated to Alexa 488 (green, excitation maximum 490 nm and emission maximum 525 nm, Santa Cruz Biotechnology) for 20 min at room temperature in the dark. Afterwards, they were identified and counted; the data are shown as bar graphs. Following AFM experiments, captured CTCs were further stained using 4′,6-diamidino-2-phenylindole (DAPI, blue, excitation maximum 377 nm and emission maximum 420 nm, Fisher Scientific) and anti-human monoclonal CK^+^ antibodies conjugated to R-Phycoerythrin (red, ﻿excitation maximum 496 nm and emission maximum 575 nm, BD Biosciences) for 30 min at room temperature in the dark to verify that EpCAM+ captured cells represent CTCs. All glass slides used in the identification/characterization of CTCs were disposed of within 3–4 h.

The background was investigated by immunostaining captured cells using anti-human monoclonal CD45^+^ (a common WBC antigen) antibodies conjugated to allophycocyanin (red, excitation maximum 650 nm and emission maximum 660 nm, Santa Cruz Biotechnology) for 20 min at room temperature in the dark. Cells were then counted in representative fields of view, and the data are presented in bar graphs as captured WBCs per mm^2^ of channel areas. The purity of the device was then calculated as the ratio of captured CTCs/PC3 cells to the total number of captured cells, including WBCs.

Furthermore, WBCs of cancer patient samples were used to verify by staining that they were negative for EpCAM staining but positive for DAPI and CD45 staining. In an additional work, flow cytometry was used to investigate the CD45+/EpCAM−/PSA−/PSMA− characteristics of WBCs.

### AFM force measurements

After peeling off the PDMS, immunostained cancer cells (CTCs or PC3 cells) on glass slides were further transferred onto the AFM stage for nanomechanical characterization. A custom-made PDMS gasket was used as the “AFM liquid cell” to hold the cell medium. Here Leibovitz medium (L-15, Sigma-Aldrich) was used to adjust the pH when cells were outside the incubator^47^. The AFM measurements were then performed with Life Sciences AFM (LS-AFM) from AFM Workshop (Signal Hill, CA, USA), which was paired with an inverted fluorescence microscope. Prior to cell indentation measurements, the sensitivity of the photodiode was calibrated on areas where the glass surface was cell-free. Then an ~6-µm-diameter colloidal silicon nitride spherical tip (NanoAndMore GmbH, Germany) with a nominal spring constant of 0.08 N/m was positioned above the center of a single captured intact cancer cell. Here unmodified AFM tip surfaces were used as a reference substrate to measure the mechanical properties of the cancer cells. Afterward, force measurements were performed in field-of-view (FV) mode, in which the approach–retraction process was repeated over a 10-μm^2^ cell surface area at resolutions of either 8 × 8 pixels (for CTCs) or 4 × 4 (for PC3 cells) per FV image (pixel sizes of 0.156 and 0.625 μm, respectively). In this way, at the single-cell level, we were able to obtain statistically significant information regarding the elasticity, deformation, and adhesiveness of cells. The measurements were performed under a constant applied loading force of approximately 6–12 nN for CTCs and 2 nN for PC3 cells, reflecting cell surface indentations of approximately 0.2–0.4 and 1.5 μm, respectively. The approach and retraction velocities of the tip were set at ~4 μm/s. Force curves with unclear approaches and/or retraction curves were excluded from the analysis. The force curves on all the pixels of FV images were analyzed with our own software^47–49^ to obtain the distribution of the Young’s moduli, cell surface deformations, adhesion forces, and work of detachment. As such, for the elasticity measurements, the Hertz model of contact mechanics was used, where the model considers the deformation of an infinite elastic half-plane (the cell surface) being compressed by a sphere indenter of radius *R* (the AFM tip):

3 $$F = \frac{4}{3}\frac{E}{{(1 - {\it{v}}^2)}}(\sqrt R )(\delta ^{3/2})$$

where *F* is the loading force, *E* is the Young’s modulus (i.e., elasticity modulus) of the cell, *δ* is the cell surface indentation, and *ν* is the Poisson ratio of the cell, which was set at 0.5 when assuming cell incompressibility. The Young’s moduli were then extracted by fitting the indentation curves to a classic Hertz model of contact mechanics (Eq. 3), where the Hertzian fit range was chosen to be within the elastic region of cell surface indentation (~100–200 nm for CTCs and 400 nm for PC3 cells). For the adhesion measurements, the maximum adhesion forces were defined from each data point in the retraction curves and were assigned as minima if they were less than their 5 nearest neighbors to the right and left. A value of 10 pN was chosen as the cut-off force due to experimental noise during measurements. For the work of detachment measurements, a trapezoidal integral was used to calculate the area confined between the zero-force line and the retraction curve. Origin software (OriginLab Inc., USA) was used to evaluate the arithmetic mean and the standard deviations of the histograms. As a proof of principle, AFM measurements were carried out on five CTCs (three with localized cancer background and two with metastatic cancer background) and five PC3 cells.

### Statistical analysis

Statistical analysis was performed with the Origin software using two-sample *t* test analysis to evaluate size differences of CTCs of localized and metastatic backgrounds. A *P* value of <0.05 was considered statistically significant.

## Supplementary information

Supplementary file.

Graphical Abstract

Editorial Summary
