# Supplementary material for: AFM-compatible microfluidic platform for affinity-based capture and nanomechanical characterization of circulating tumor cells
Source: Microsyst Nanoeng. 2020 Mar 23;6:20. doi: 10.1038/s41378-020-0131-9 (PMC8433216; doi:10.1038/s41378-020-0131-9)
Supplement: Supplementary file 1 — Supplementary file. [file 41378_2020_131_MOESM1_ESM.pdf]

Supplementary file

AFM-compatible microfluidic platform for affinity-based capture and nanomechanical characterization of circulating tumor cells

Muhammedin Deliorman[1], Farhad K. Janahi[2,†], Pavithra Sukumar[1,†], Ayoub Glia[1,5], Roaa Alnemari[1,‡], Samar Fadl[3,‡], Weiqiang Chen[4,5], and Mohammad A. Qasaimeh[1,5,*]

[1] Division of Engineering, New York University Abu Dhabi, P.O. Box 129188, Abu Dhabi, UAE

[2] Mohammed Bin Rashid University of Medicine and Health Sciences, P.O. Box 505055, Dubai, UAE

[3] Division of Science, New York University Abu Dhabi, P.O. Box 129188, Abu Dhabi, UAE

[4] Department of Biomedical Engineering, New York University, 10003 NY, USA

[5] Department of Mechanical and Aerospace Engineering, New York University, 10003 NY, USA

[*] E-mail: mohammad.qasaimeh@nyu.edu

[†, ‡] These authors contributed equally

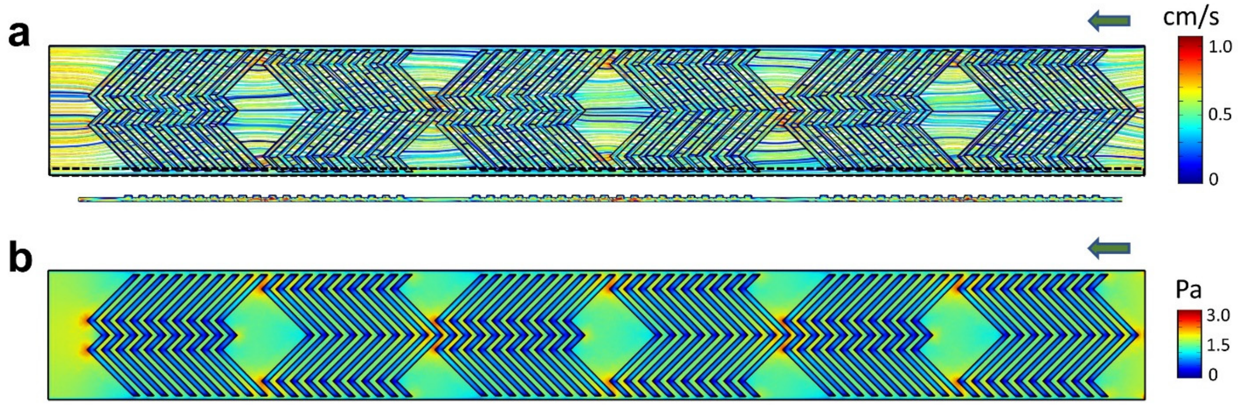

Figure S1 (**a**) The 3D numerically simulated flow profile within the channel indicates high degree of mixing at 20 µL/min flow rate due to microvortices generated by the staggered HB grooves. The green arrow shows the direction of the flow. 3D velocity streamlines shown in the bottom panel proves high mixing. (**b**) The estimated shear stresses within the channel (<2 Pa).

Supplementary file

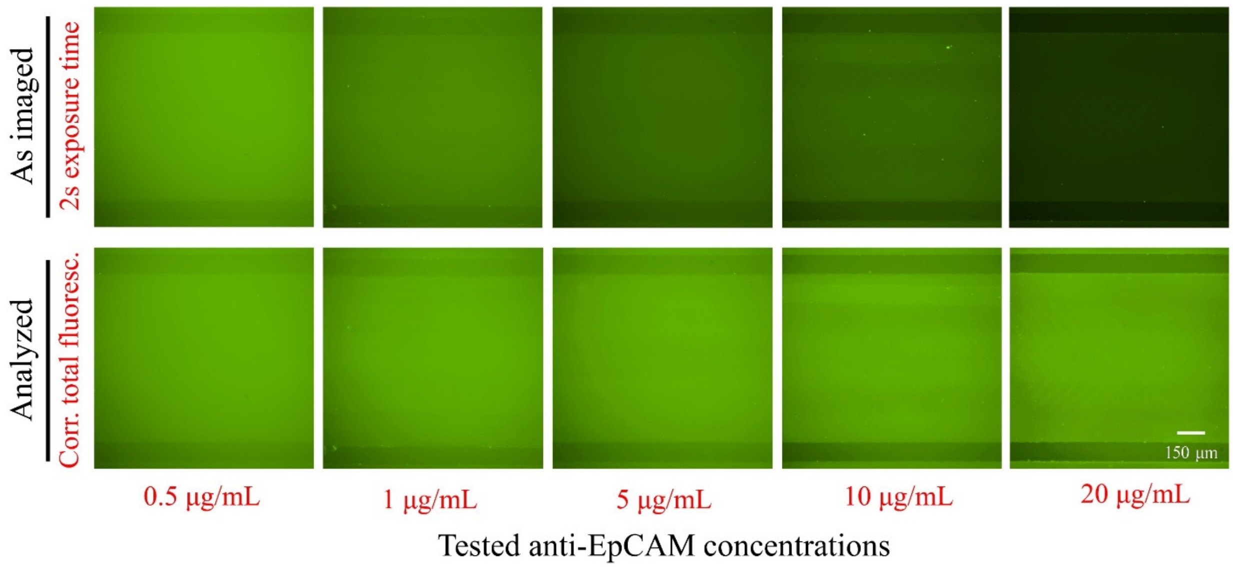

Figure S2 The surface concentrations and orientations of anti-EpCAM antibodies (green) immobilized on glass substrates of microfluidic devices were verified by labeling their antigen binding sites (located in their arms) with NHS-fluorescein. An algorithm was used to calculate the corrected total fluorescence of images, where it scaled and shifted each pixel value of the input image (control, 0.5 μg/mL anti-EpCAM antibody concentration) in order to match its mean and standard deviation to those of target images (corrected).

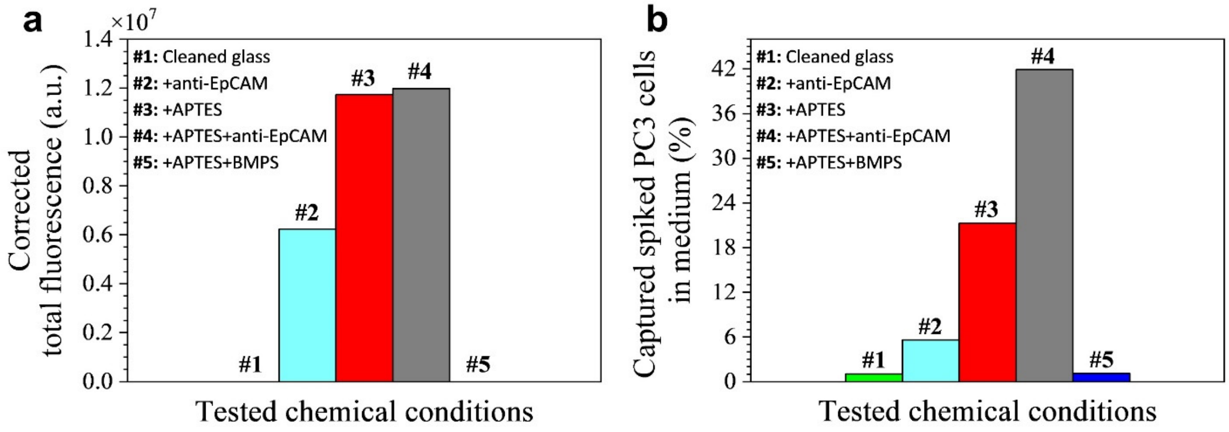

Figure S3 Dependence of anti-EpCAM antibody densities and PC3 cell capture efficiencies on chemical steps involved in the glass functionalization of microfluidic devices. Following each step in APTES-BMPS chemistry and anti-EpCAM antibody activation, glass substrates of the microfluidic devices were characterized using (**a**) NHS-fluorescein and (**b**) PC3 cells spiked in culture medium. In antibody characterization experiments, anti-EpCAM antibodies were immobilized at 10 μg/mL concentrations. In cell capture experiments, PC3 cells were spiked in culture medium at 1000 cells/mL concentrations and 1 mL of cell suspension was introduced in 10 μg/mL anti-EpCAM antibody-activated microfluidic devices at 20 mL/min flow rates.

Supplementary file

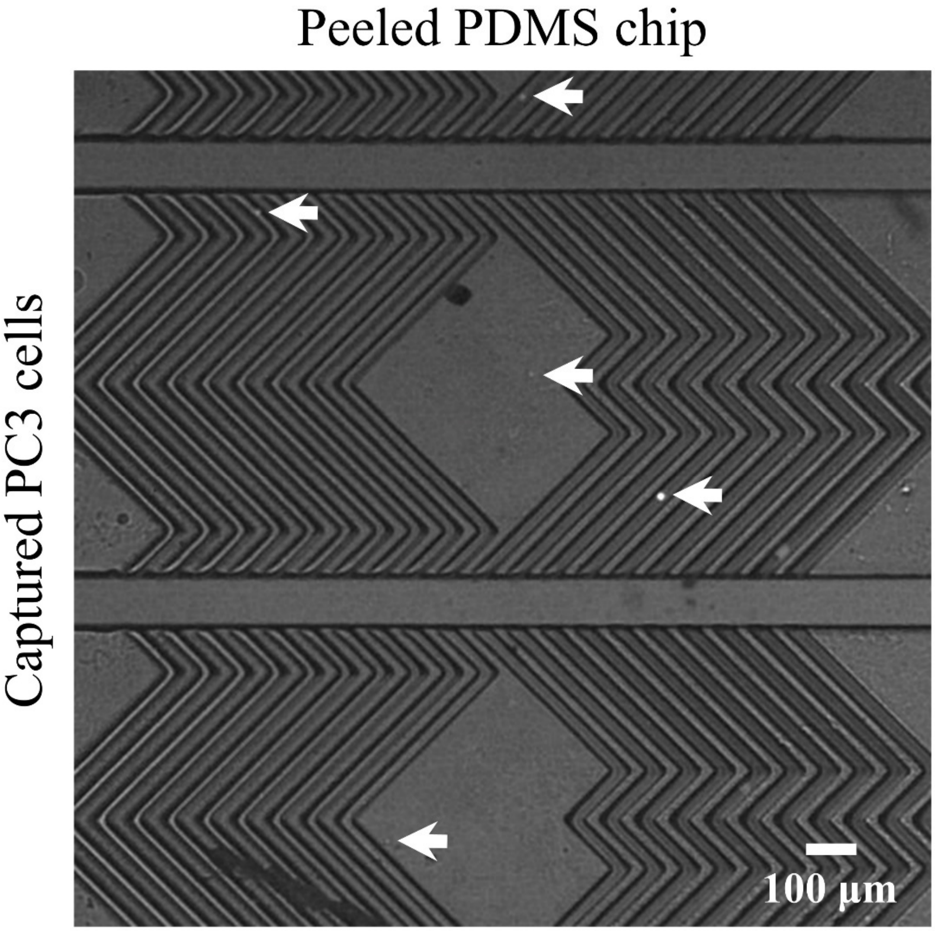

Figure S4 Following peeling off the PDMS chip from glass slide, PC3 cells (white dots) on the PDMS surfaces were mostly captured through physically adsorbed antibodies and contributed to total capture efficiency of the microfluidic device at ~7%.

Supplementary file

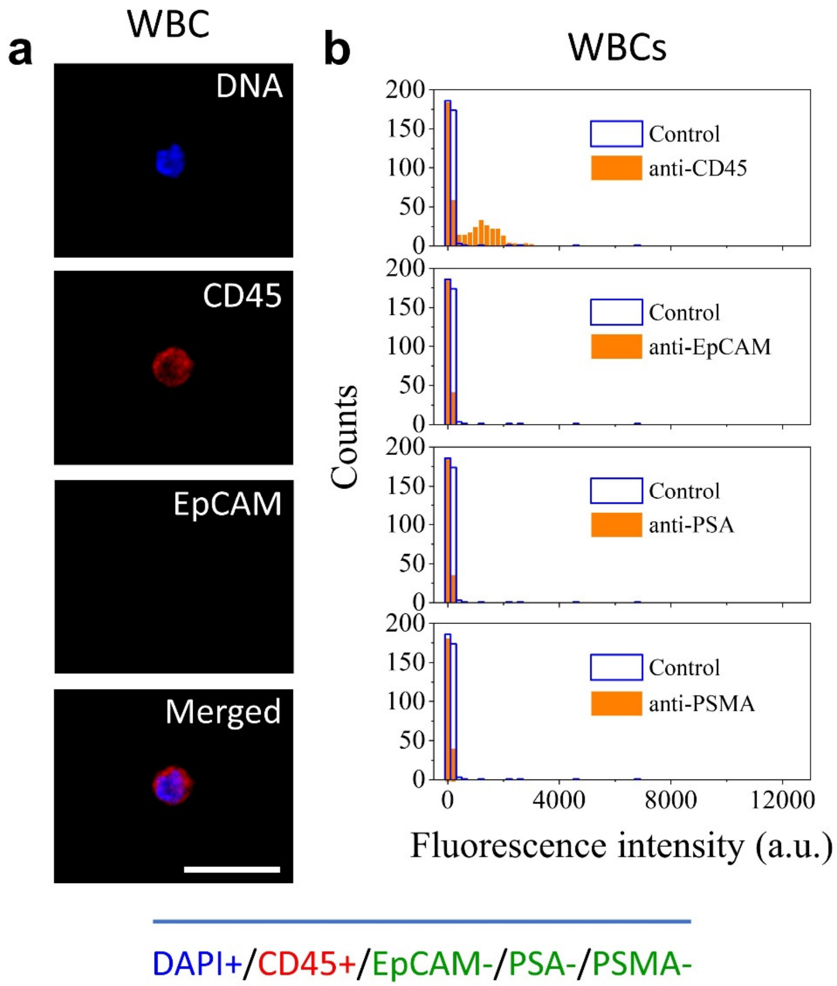

Figure S5 (**a**) Fluorescent images of a captured patient WBC (obtained through Ficoll density gradient separation) proved positive to immunostaining of their nucleus (blue, DAPI+), CD45 (red, CD45+) and negative to staining EpCAM expressions. Scale bar is 20 µm. (**c**) As revealed by flow cytometry, WBCs did not bind to anti-EpCAM, anti-PSA, and anti-PSMA, but only to anti-CD45 antibodies confirming their CD45+/ EpCAM-/PSA-/PSMA- characteristics.

Supplementary file

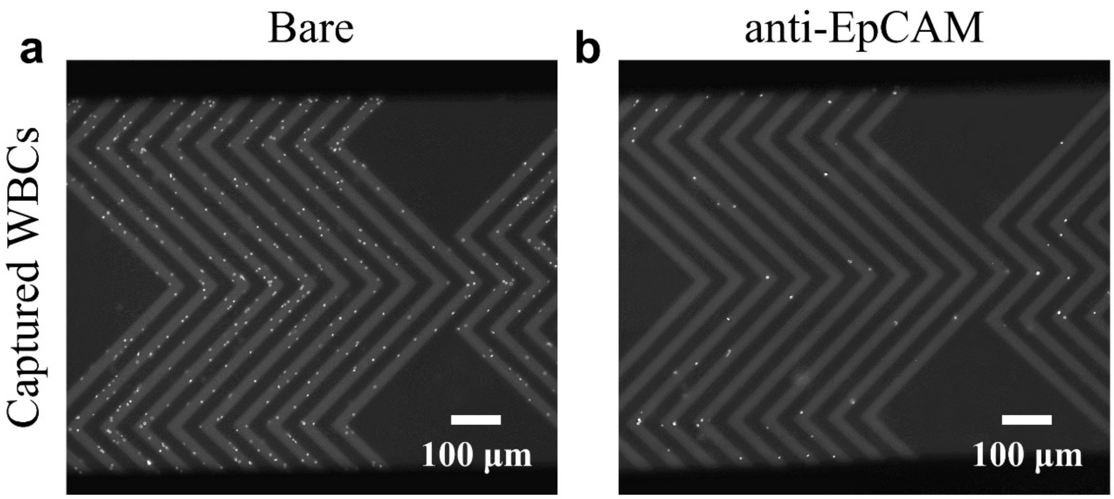

Figure S6 Comparison of the number of non-specifically captured WBCs (white dots) on cleaned and 10 µg/mL anti-EpCAM antibody (Ab)-activated glass substrates of the microfluidic device after washing the channels with PBS at 20 mL/min flow rates.

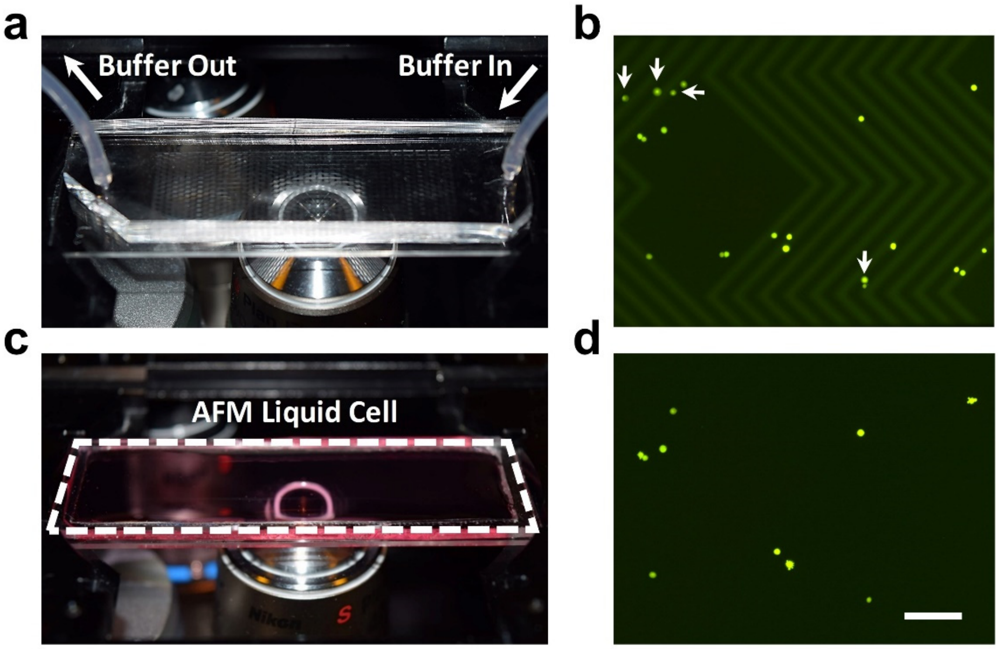

Figure S7 Dissembling the microfluidic capture device for downstream AFM measurements on captured intact cancer cells. (**a**) Following cell capture experiments, microfluidic channels were washed with PBS in order to remove any unattached cells. (**b**) After washing, captured PC3 cells were identified within the channels and counted. (**c**) The PDMS chip was then removed (peeled off) from the glass slide and a custom-made AFM liquid cell was used to confine the cell culture medium. (**d**) Further imaging verified that majority (>60%) of the captured cells remained on the glass substrates for their mechanical characterization using AFM. Evidenced by the change in focus, some PC3 cells (e.g. white arrows) were captured on PDMS chip surfaces. Scale bar is 160 µm.

Supplementary file

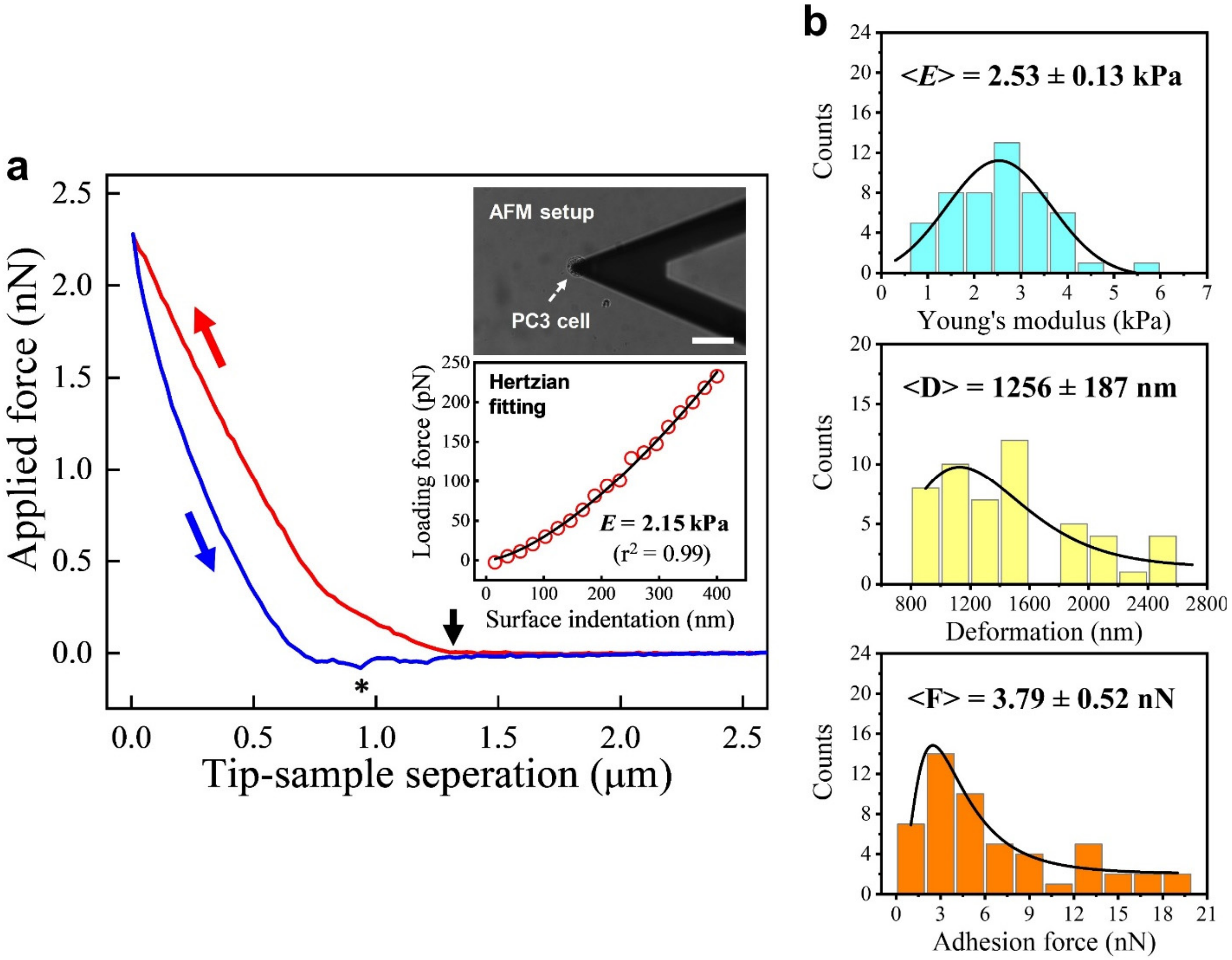

Figure S8 AFM analysis of the microfluidic-captured single intact PC3 cells. (**a**) Representative AFM force curve demonstrates the interaction of the AFM tip with a single captured intact PC3 cell, where the tip's approach to and retraction from the cell surface are represented with the red and blue arrows, respectively. The black arrow indicates the tip contacting the cell surface and the * shows the maximum adhesion force between the tip and the cell surface. Top inset: Micrograph shows the AFM tip positioned above the center of a single PC3 cell for its follow up nanomechanical characterizations (Scale bar: 20 μm). Bottom inset: Loading force versus surface indentation curve (open red circles) shows the data fit (solid black lines) to Hertz model. (**b**) Histograms of the distribution of all Young's moduli, *E*, deformations, D, and maximum adhesion forces, F, as measured for PC3 cells (n = 5). Solid black lines are data fit to lognormal or Gauss probability density functions. Data represent mean ± S.D.
