# Supplementary figures and images for: AFM-compatible microfluidic platform for affinity-based capture and nanomechanical characterization of circulating tumor cells
Source: Microsyst Nanoeng. 2020 Mar 23;6:20. doi: 10.1038/s41378-020-0131-9 (PMC8433216; doi:10.1038/s41378-020-0131-9)

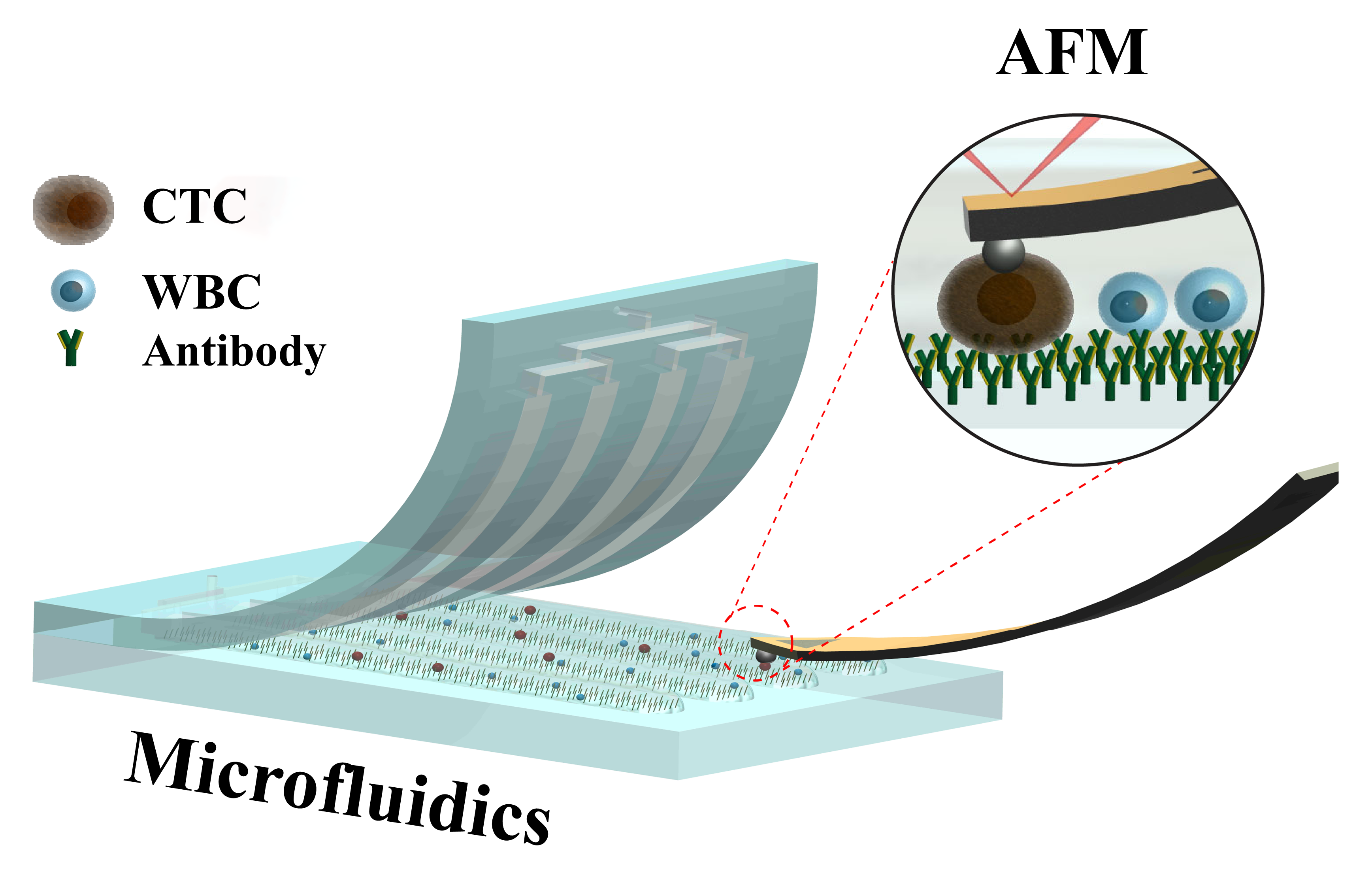

Supplement: Supplementary file 2 — Graphical Abstract [file 41378_2020_131_MOESM2_ESM.tif]
