# Supplementary material for: AFM-compatible microfluidic platform for affinity-based capture and nanomechanical characterization of circulating tumor cells
Source: Microsyst Nanoeng. 2020 Mar 23;6:20. doi: 10.1038/s41378-020-0131-9 (PMC8433216; doi:10.1038/s41378-020-0131-9)
Supplement: Supplementary file 3 — Editorial Summary [file 41378_2020_131_MOESM3_ESM.docx]

# *Microsystems & Nanoengineering*

Cancer research: Investigating the drivers of metastasis

A microfluidic platform allows for the capture and characterization of circulating tumor cells, a major driver of invasive cancer. Circulating tumor cells (CTCs) break off from the original tumor and invade distant sites; however, they’re hard to isolate and analyze because their concentration in blood is so low. Mohammad Qasaimeh, of New York University Abu Dhabi, and his team of UAE and US scientists created a device with microfluidic channels, that traps cancer cells using targeted antibodies that are bound to the device’s glass surface. Once captured, a high-resolution microscopy technique can measure the cells’ mechanical attributes. The team tested the device on prostate cancer cells and describe that CTCs from metastatic cancers show differences in elasticity, deformability, and adhesion when compared to localized cancers. This technique offers a promising tool to investigate cancer metastasis.

Related article manuscript number: MICRONANO-00997R

Article title: An AFM-compatible microfluidic platform for affinity-based capture and nanomechanical characterization of circulating tumor cells

Corresponding author and affiliation/s: Mohammad A. Qasaimeh, New York University Abu Dhabi, United Arab Emirates

**About your Editorial Summary — please read**

**Before approving this Editorial Summary, please carefully check that (1) the summary text lists the correct author(s) and (2) the spelling and order of all author names and affiliations are correct.**

This **Editorial Summary** is based on your manuscript that was recently accepted for publication in *Microsystems & Nanoengineering*. It provides a non-specialist audience with a synopsis of your key research outcomes and conclusions. This value-added service provided by Springer Nature is designed to raise interest in your research across the broader community.

Springer Nature will publish the summary on the journal’s website, and it will be freely available under a under the CC BY licence (Creative Commons Attribution v4.0 International Licence) (see the journal website for details). We encourage you to re-use the summary to bring attention to your research; for example, you can host it on your own website and share it via social-networking platforms. Please attribute the summary to *Microsystems & Nanoengineering* and your article (e.g. by providing a link to your article) and do not make derivatives.

Please note that to maximise the usefulness of these summaries they must follow several stringent guidelines:
-- Spelling, punctuation and style are set according to *Nature* editorial guidelines. As this summary is aimed at non-expert readers, some concepts and technical terms will be simplified.
-- Total length must be no more than 135 words. It is likely that not all points in the paper will be covered.
-- The first sentence must be no more than 280 characters, including spaces, to allow use on microblogging sites.
-- The headline must consist of a brief generic subject identifier followed by a short description. No more than 10 words in total.

Please contact the editorial office ([mems_nano@mail.ie.ac.cn](mailto:mems_nano@mail.ie.ac.cn)) immediately with corrections should you find any factual errors in this Editorial Summary.
